# Bevacizumab significantly increases the risks of hypertension and proteinuria in cancer patients: A systematic review and comprehensive meta-analysis

**DOI:** 10.18632/oncotarget.18190

**Published:** 2017-05-23

**Authors:** Tingting Zhao, Xiaonan Wang, Tingting Xu, Xiaodong Xu, Zhihong Liu

**Affiliations:** ^1^ National Clinical Research Center of Kidney Diseases, Jinling Hospital, Nanjing University School of Medicine, Nanjing, Jiangsu, 210002, China

**Keywords:** bevacizumab, chemotherapy, hypertension, proteinuria, cancer

## Abstract

Published data regarding the overall risks and incidence of hypertension and proteinuria associated with bevacizumab were still unclear. To quantify the precise risks and incidence, we performed this comprehensive meta-analysis of 72 published clinical trials including 21902 cases and 20608 controls. The overall incidence, risk ratios (RRs), and 95% confidence intervals (95% CIs) were calculated using a fixed or random-effect model based on the heterogeneity. The incidence of all-grade and high-grade hypertension were 25.3% (95% CI: 21.5%−29.5%) and 8.2% (95% CI: 7%−9.8%) for patients treated with bevacizumab. And the incidence of all-grade and high-grade proteinuria were 18% (95% CI: 11.7%−26.6%) and 2.4% (95% CI: 1.8%−3.2%), respectively. Compared with controls, bevacizumab significantly increased the risks of all-grade (RR: 3.595, 95% CI: 2.952−4.378) and high-grade hypertension (RR: 5.173, 95% CI: 4.188−6.390). Obviously increased risks of all-grade (RR: 3.369, 95% CI: 2.492−4.556) and high-grade proteinuria (RR: 5.494, 95% CI: 3.991−7.564) were also observed. In the subgroup analysis, the risks of hypertension and proteinuria may significantly vary with bevacizumab dosage, cancer types and concomitant drugs. Whereas, no obvious difference were discovered when stratified based on phase of trials, age of patients, treatment line and duration. So, close monitor and effective management were highly recommended for the safe use of bevacizumab.

## INTRODUCTION

Tumor angiogenesis mediated by vascular endothelial growth factor (VEGF) plays a pivotal role in the growth, invasion, and metastasis of tumor [1–3]. So, the VEGF signaling pathway has been a major focus in current cancer treatment [3, 4]. Bevacizumab (Avastin, Genentech, South San Francisco, CA), a recombinant humanized monoclonal antibody against VEGF, has been widely used in the treatment of various cancers, including colorectal cancer, breast cancer, lung cancer, renal cell cancer, ovarian cancer, pancreatic cancer, gastric cancer, glioblastoma and so on [5–18].

Similar to other angiogenesis inhibitor, bevacizumab may also lead to substantial adverse effects, such as nausea, fatigue, diarrhea, hemorrhage, thrombosis, wounding-healing complications and renal toxicities [19]. Hypertension and proteinuria are the dominant adverse effects for renal toxicities [20]. Previous studies have demonstrated that the RRs of all-grade proteinuria for patients administered bevacizumab at 2.5 mg and 5 mg/kg/week were 1.4 (95% CI: 1.1–1.7) and 2.2 (95% CI: 1.6–2.9), respectively, and the RRs of all-grade hypertension for different dosage were 3.0 (95% CI: 2.2–4.2) and 7.5 (95% CI: 4.2–13.4), respectively [20].

High-grade hypertension and proteinuria (grade 3–4), especially hypertensive crisis and nephrotic syndrome, may cause obvious cardiovascular damage and renal failure. Those life-threatening consequence would limit the dose of bevacizumab, thereby reducing its efficacy [21]. The incidence of high-grade proteinuria for patients accepted bevacizumab varied considerably, ranging from 0.3% in a breast cancer study to 15.5% in an renal cell cancer study [8, 22]. The similar variation also exited for the incidence of high-grade hypertension, ranging from 0.7% in a colorectal cancer study to 60% in an lung cancer study [23, 24]. Due to the limited number of patients available in each clinical trial and a great deal of large sample size randomized controlled trials (RCTs) have been carried out, the power of the previous meta-analysis to fully elucidate the risks and incidence of proteinuria and hypertension with bevacizumab was immature [20, 21, 25]. Therefore we performed this systematic meta-analysis including all available published RCTs focused on different subgroups to estimate the overall risks and incidence of hypertension and proteinuria associated with bevacizumab.

## RESULTS

### Search results

**Figure 1:**
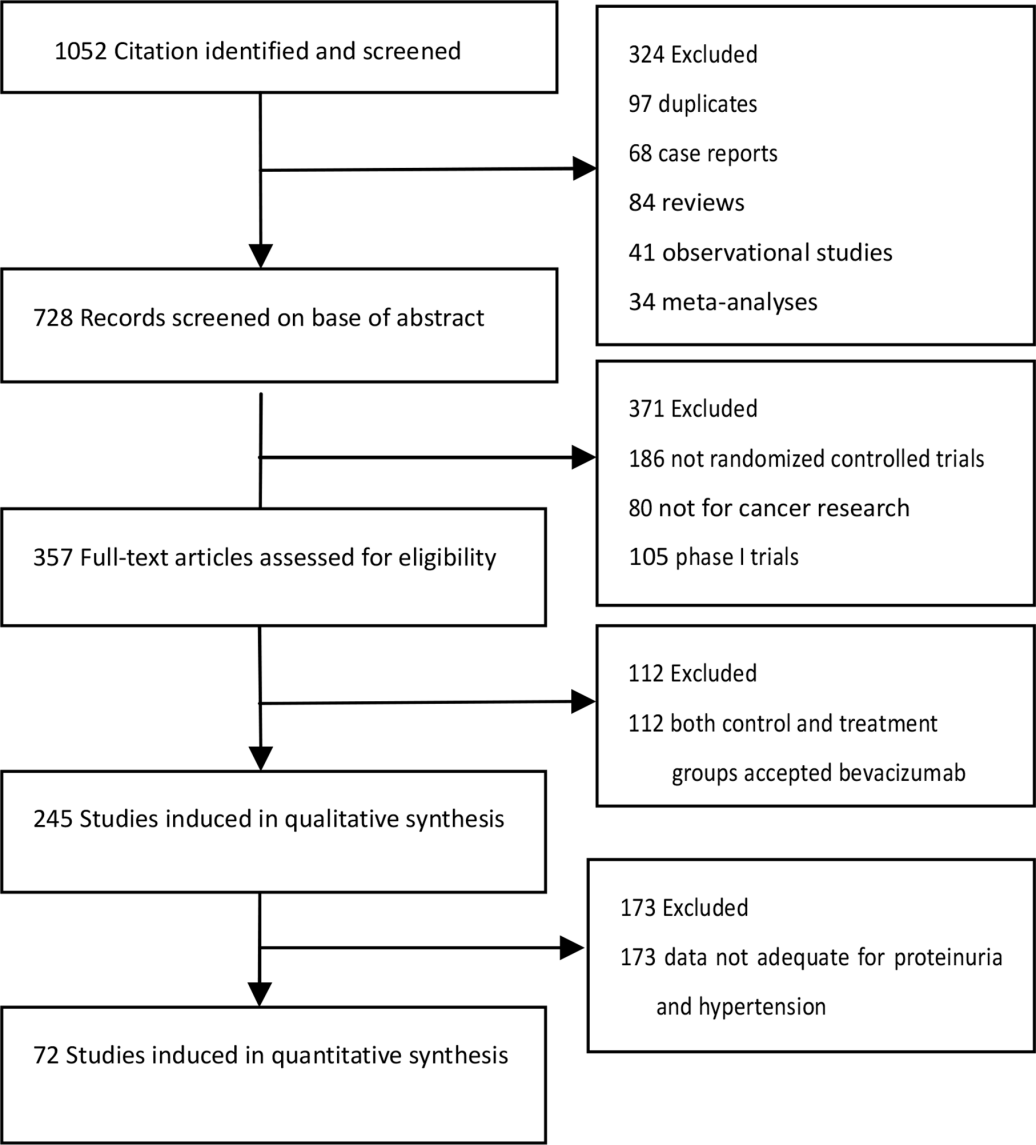
Selection process for randomized–controlled trials (RCTs) included in the meta-analysis

Over 1052 clinical literatures relevant to the search terms were obtained. After selection by title screening, clinical data quality check, and abstract review, a total of 72 eligible studies were identified for analysis (Figure 1), which contained 21902 cases and 20608 controls. All of the patients enrolled had adequate hepatic, renal, and hematologic function, and the baseline Eastern Cooperative Oncology Group (ECOG) status for most of the patients was between 0 and 1. These trials included 23 phase II and 49 phase III studies, and the characteristics of selected studies were summarized in Supplementry Table 1.

All of the studies included twenty-one colorectal cancer [5, 24, 26–45], fifteen breast cancer [6, 22, 46–58], fifteen lung cancer [7, 23, 59–71], three renal cell cancer [8, 72, 73], two pancreatic cancer [10, 74], four ovarian cancer [9, 75–77], two gastric cancer [11, 78], two glioblastoma [12, 79], two lymphoma [13, 80], two melanoma [14, 81], one malignant mesothelioma [15], one prostate cancer [16], one cervical cancer [17] and one leiomyosarcoma [18]. The level of hypertension and proteinuria were assessed and recorded according to CTCAE version 1, 2, 3 or 4. Version 1 was used in 2 trials, version 2 was used in 11 trials, version 3 was used in 39 trials, Version 4 was used in 6 trials, and the remainder 14 trials did not specify the version. In addition, 24 trials were treated with low-dose (2.5 mg/kg/week) and 42 trials were treated with high-dose (5mg/kg/week) bevacizumab. Other six three-arm studies were also included: two arms of different dosage levels of bevacizumab and one arm of control [38, 53, 62, 64, 67, 73] (Supplemtry Table 1). The quality of all the trials was acceptable.

### Hypertension

#### High-grade hypertension

**Figure 2:**
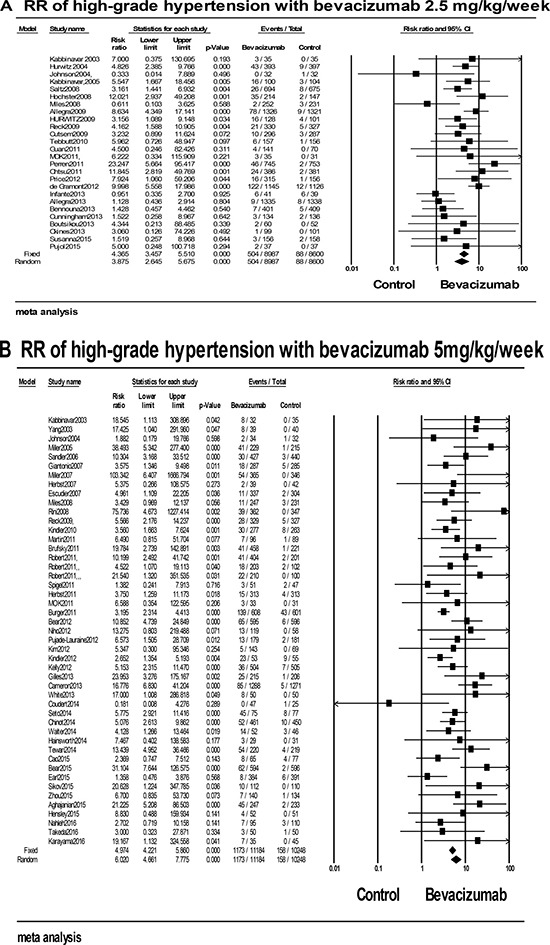
RRs of high-grade hypertension for cancer patients who received (A) low-dose and (B) high-dose bevacizumab compared with controls

A total of 39019 patients from 66 RCTs with available high-grade hypertension data were included for analysis [5–18, 22–24, 26–30, 32–39, 41–43, 45–57, 59–68, 70–79, 81]. Our results demonstrated that the summary event rate was 8.2% (95% CI: 7%–9.8%, Supplementry Table 2) in a random-effect model for the patients administered with bevacizumab. The RR was 5.173 (95% CI: 4.188–6.390) compared with controls, indicating an obviously increased risk of high-grade hypertension with bevacizumab. Further stratified analysis based on bevacizumab dosage, tumor types, and phases of trials, treatment lines, concomitant drugs, age of patients and treatment duration were conducted to explore the real relationships between the increased risks and various clinical characters. In the stratified analysis by the dosage of bevacizumab, the RR of high-grade hypertension for low-dose bevacizumab was 3.875 (95% CI: 2.645–5.675), and the RR for high-dose was 6.020 (95% CI: 4.661–7.775) as shown in Figure 2A and 2B. A significant difference (*P* = 0.033) was existed between the low and high dosage, suggesting that the risk may be dose-dependent. In the subgroup analysis by caner types, although obviously increased risks were observed in all types, the RR significantly varied (*P* = 0.039), with the highest RR for rental cancer 13.074 (95% CI: 2.631–64.96) and the lowest RR for pancreatic cancer 3.472 (95% CI: 1.804–6.679) (Supplementry Table 2). We also conducted subgroup analysis base on treatment line and phase of trials. No significant difference was observed between patients in phase II and phase III trials (RR: 3.387 VS. 5.874, *P* = 0.155), which was similar to the result between per-treated patients and native-treated patients (RR: 5.182 VS. 5.086, *P* = 0.728, Supplementry Table 2). In addition, subgroup analysis stratified based on concomitant drugs was also performed, the incidence of high-grade hypertension varied from 6.1% to 10.9%. But, no significant difference was observed (P = 0.808). Besides, in the subgroup analysis by the length of bevacizumab treatment duration, patients with long treatment had the RR of 7.045 (95% CI: 4.556–10.894), and others in short treatment had the RR of 4.192 (95% CI: 2.958–5.942). But, no obvious difference was obtained between the short and long treatment (*P* = 0.359, Supplementry Table 2). Finally, subgroup analysis base on the age of patients was also conducted, but no significant difference for the RR was observed between patients < 60 and ≥ 60 years (RR: 5.774 VS. 3.690, *P* = 0.08).

### All -grade hypertension

**Figure 3:**
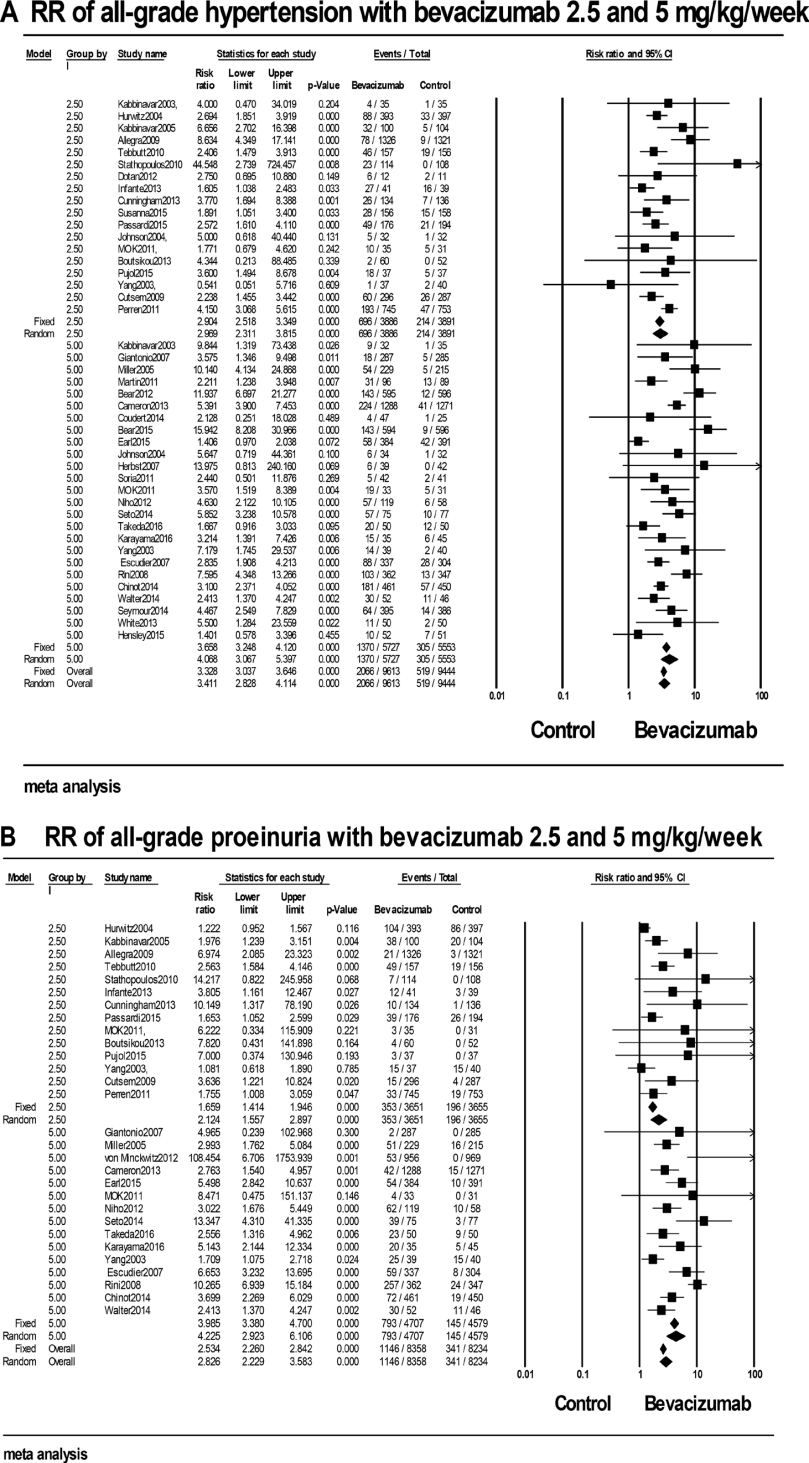
RRs of (**A**) all-grade hypertension and (**B**) all-grade proteinuria for cancer patients who received low-dose and high-dose bevacizumab compared with controls.

A total of 19057 patients from 39 RCTs with available all-grade hypertension data were included for the analysis [5, 7, 8, 12, 14, 18, 22, 23, 26, 30–32, 37–41, 44–47, 49, 50, 52, 55, 59, 60, 62–66, 69, 72–74, 76, 79, 80]. For the patients accepted bevacizumab, our result demonstrated that the incidence was 25.3% (95% CI: 21.5%-29.5%, Supplementry Table 3) calculated in a random-effect model. The RR was 3.59 (95% CI: 2.952–4.378) compared with controls, indicating an obviously increased risk for all-grade hypertension with bevacizumab. In the subgroup analysis by the dosage of bevacizumab, the RR for low-dose bevacizumab was 2.969 (95% CI: 2.311–3.815) and for high-dose was 4.068 (95% CI: 3.067–5.397) as shown in Figure 3A. Whereas, no significant difference was obtained between the low and high dosage of bevacizumab (*P* = 0.991). In the stratified analysis by caner types, obviously increased risks were observed in all cancer types, with the highest RR for breast cancer 5.119 (95% CI: 2.415–10.849) and the lowest for pancreatic cancer 2.238 (95% CI: 1.455– 3.342). But there were no significant difference between various cancer types (*P* = 0.943, Supplementry Table 3). We also conducted subgroup analysis base on treatment line and phase of trials. No significant difference was observed between patients in phase II and phase III trials (RR: 3.134 VS. 3.795, *P* = 0.438), which was similar to the result between per-treated patients and native-treated patients (RR: 3.662 VS. 3.219, P = 0.671). In addition, subgroup analysis stratified based on concomitant drugs was also performed, with the highest RR 7.686 (95% CI: 0.537–109.921) in conjunction with irinotecan and the lowest RR 2.350 (95% CI: 1.645–3.358) used in combination with gemcitabine (Supplementry Table 3). But, no significant different was observed between various concomitant drugs (*P* = 0.126). Besides, in the stratified analysis by the length of treatment duration, patients with long treatment had the RR of 4.173 (95% CI: 2.641–6.592), and others in short treatment had the RR of 5.496 (95% CI: 3.690–8.187). But, no obvious difference was obtained between the short and long treatment (*P* = 0.934, Supplementry Table 3). Finally, subgroup analysis base on the age of patients was also conducted, but no significant difference was observed for the RR of all-grade hypertension between patients < 60 and ≥ 60 years (RR: 2.848 VS. 3.163, *P* = 0.225).

### Proteinuria

#### High-grade proteinuria

**Figure 4:**
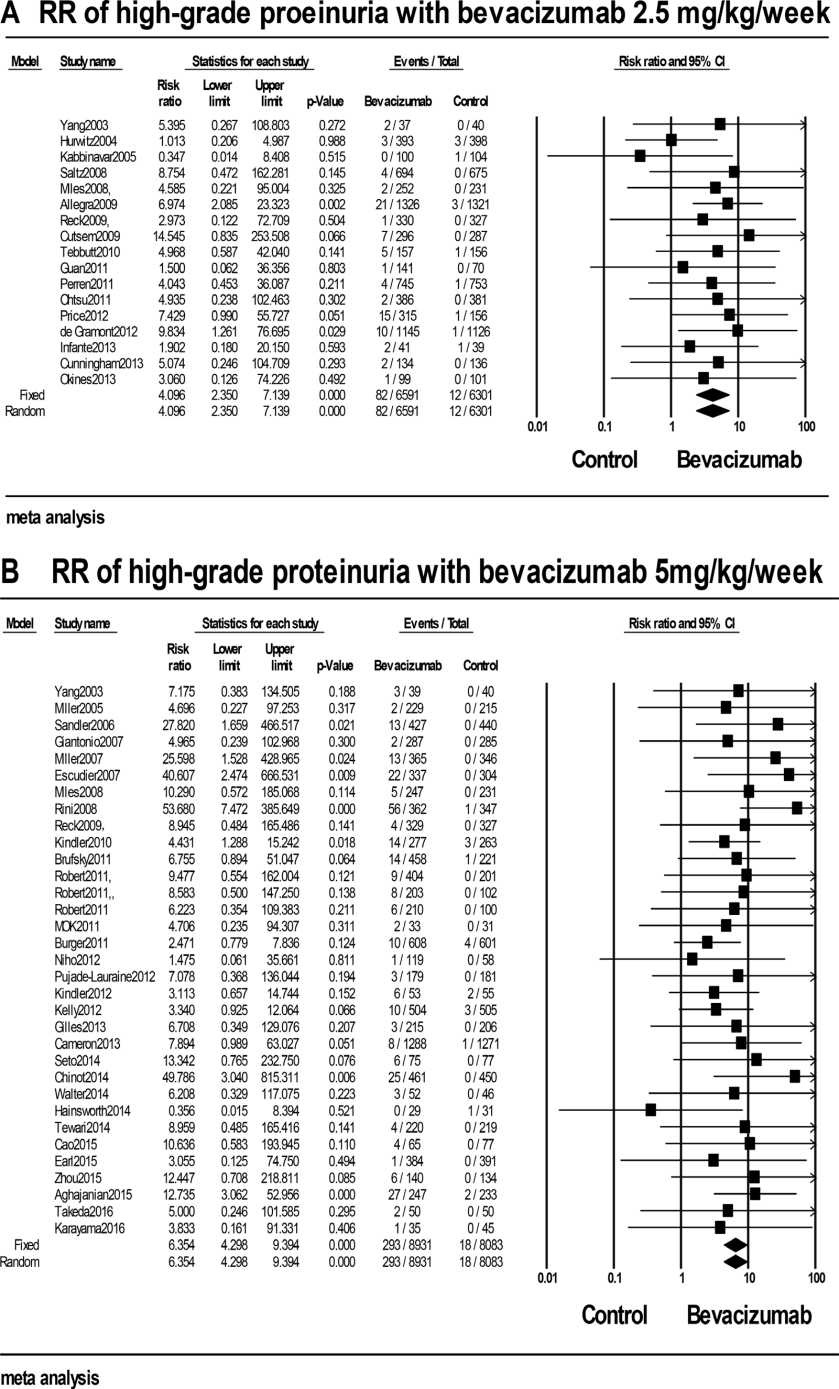
RRs of high-grade proteinuria for cancer patients who received (**A**) low-dose and (**B**) high-dose bevacizumab compared with controls.

A total of 29906 patients from 45 RCTs with available high-grade proteinuria data were included for the analysis [7–13, 15–17, 22, 23, 26, 28–30, 32, 33, 35, 37, 39, 41–43, 45, 48, 49, 51, 53–56, 63–65, 67, 68, 71–79]. Among the patients received bevacizumab, our results demonstrated that the incidence was 2.4% (95% CI: 1.8%-3.2%) calculated in a random-effect model (Supplementry Table 2). For trials excluding rental cancer, the summary incidence was 2.2% (95% CI: 1.7%-2.8%), suggesting that no significant difference was discovered with and without rental cancer (*P* = 0.427). Compared with controls, the RR for high-grade proteinuria was 5.494 (95% CI: 3.991–7.564), indicating an obviously increased risk with bevacizumab. In the stratified analysis by the dosage, the incidence of high-grade proteinuria with low-dose bevacizumab was 1.4% (95% CI: 0.9%–2.1%), and the incidence of high-dose was 3.2% (95% CI: 2.3%–4.4%) as shown in Figure 4A and 4B. A significant difference (*P* = 0.012) was existed between the low and high dosages of bevacizumab, suggesting that the incidence may be dose-dependent. In the subgroup analysis by caner types, the RR significantly varied (*P* = 0.008, Supplementry Table 2), with the highest RR for rental cancer 22.786 (95% CI: 6.347–81.804) and the lowest for gastric cancer 3.933 (95% CI: 0.437–35.412). Stratified analysis base on treatment line and phase of trials, no significant difference was observed for the RR between patients in phase II and phase III trials (RR: 3.181 VS. 6.206, *P* = 0.076), which was similar to the result between per-treated patients and native-treated patients (RR: 5.351 VS. 6.282, *P* = 0.977, Supplementry Table 2). In addition, subgroup analysis stratified based on concomitant drugs was also performed and the RRs significantly varied (*P* < 0.001). The highest RR was existed when in conjunction with interferonalfa 48.931 (95% CI: 9.763–245.31) and the lowest was observed when used in combination with irinotecan 1.704 (95% CI: 0.474–6.128). Besides, we did subgroup analysis according to the length of bevacizumab treatment duration. Patients with long treatment had RR of 5.786 (95% CI: 2.746–12.189), and others in short treatment had RR of 5.784 (95% CI: 3.160–10.588). But, no obvious difference was obtained between the short and long time treatment (*P* = 0.496, Supplementry Table 2). Finally, subgroup analysis base on the age of patients was also conducted, but no significant difference was observed for the RR of high-grade proteinuria between patients < 60 and ≥ 60 years (RR: 5.618 VS. 4.401, *P* = 0.606).

### All -grade proteinuria

A total of 16592 patients from 27 RCTs with available all-grade proteinuria data were included for the analysis [7, 8, 12, 22, 23, 26, 30, 32, 37, 39–41, 44, 45, 49, 55, 58, 60, 63–66, 72–74, 76, 79]. For the patients administered with bevacizumab, our results demonstrated that the summary event rate was 18% (95% CI: 11.7%-26.6%) calculated in a random-effect model. For trials excluding rental cancer, the incidence was 14.3% (95% CI 9.4%–21.2%, Supplementry Table 3), but no significant difference was discovered with and without rental cancer (*P* = 0.502). Compared with controls, the RR was 3.369 (95% CI: 2.492–4.556), indicating an obviously increased risk for all-grade proteinuria with bevacizumab. In the subgroup analysis by the dosage, the RR for low-dose bevacizumab was 2.124 (95% CI: 1.557–2.897), and the RR for high-dose was 4.225 (95% CI: 2.923–6.106) as shown in Figure 3B. But, no significant difference (*P* = 0.311) was existed between the low and high dosage of bevacizumab. Subgroup analysis based on caner types, the incidence of all-grade proteinuria ranged from 4.4% (95% CI: 3.2%-6.2%) for ovarian cancer to 47.1% (95% CI: 17.3%-79.1%) for rental cancer (Supplementry Table 3). But, no significant difference was discovered between various caner types (*P* = 0.065). In addition, we also conducted subgroup analysis base on treatment line and phase of trials. No significant difference was observed between patients in phase II and phase III trials (RR: 2.579 VS. 3.983, *P* = 0.397), which was similar to the result between per-treated patients and native-treated patients (RR: 3.574 VS. 2.973, *P* = 0.517, Supplementry Table 3). Stratified analysis by concomitant drugs, although significantly increased risks were observed in all concomitant drugs, no significant different were observed (*P* = 0.536), with the highest RR in conjunction with cyclophosphamide 11.515 (95% CI: 2.106–62.9) and the lowest RR in combination with taxane 2.381 (95% CI: 1.690–3.354, Supplementry Table 3). Besides, we also did subgroup analysis according to the length of bevacizumab treatment duration. Patients with long treatment had the RR of 2.893 (95% CI: 1.304–6.416), while others in short treatment had the RR of 10.14 (95% CI: 6.926–14.86). But, no obvious difference was obtained between the short and long treatment (*P* = 0.877, Supplementry Table 3). Finally, subgroup analysis base on the age of patients was also conducted, but no significant difference was observed between patients < 60 and ≥ 60 years (RR: 2.382 VS. 3.406, *P* = 0.424). Because the limited number of patients in each trial and the limited number of enrolled RCTs, more cautious should be paid when interpreting those results.

### Publication bias

**Figure 5:**
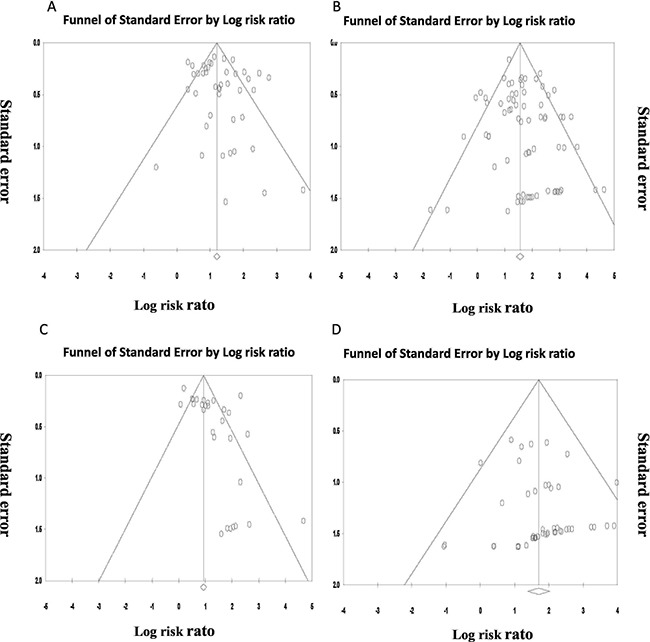
Funnel-plot standard error based on the risk ratio for relative risk of (A) all-grade hypertension and (B) high-grade hypertension (C) all-grade proteinuria and (D) high-grade proteinuria

We carried out Begg's funnel plot and Egger's test to assess the publication bias of the included studies. As shown in Figure 5, the shape of the funnel plots seemed asymmetrical in all and high-grade proteinuria analysis, indicating the existing of publication bias. Then, we performed the Egger's test to provide statistical evidence for funnel plot asymmetry. As expected, the results showed obvious evidence of publication bias for all-grade (*t* = 0.293, Z = 2.23, *P* = 0.026) and high-grade proteinuria (*t* = −0.339, Z = 3.48, *P* = 0.0005), but not for all-grade (*t* = 0.09, Z = 0.84, *P* = 0.0.40) and high-grade hypertension (*t* = 0.02, Z = 0.27, *P* = 0.79, Figure 5). A trimand-fill method developed by Duval and Tweedie was performed to adjust for this bias. No different conclusions were drawn with or without the trim-and-fill method, which indicating that our results were statistically robust [82].

## DISCUSSION

Bevacizumab has been clinically validated as a targeted agent against various cancers and also may lead to a great deal of adverse effects [19]. Because proteinuria and hypertension are vital risk factors for renal and cardiovascular events, it is particularly essential to recognize and take adequate and aggressive management to monitor and manage those risks timely and appropriately. So, our present meta-analysis systematically investigated the comprehensive association between the increased risks and incidence of proteinuria and hypertension associated with bevacizumab among different cancer patients.

Our study demonstrated that bevacizumab was associated with a significantly increased risks for all-grade (RR: 3.369, 95% CI: 2.492–4.556) and high-grade proteinuria (RR: 5.494, 95% CI: 3.991–7.564) in comparison with controls. In clinical, angiotensin receptor blockers and angiotensin-converting enzyme inhibitors were normally used to manage bevacizumab related proteinuria. In addition, bevacizumab was recommended to temporarily suspended for patients with urine protein excretion more than 2 g/24h, and resumed when it was less than 2 g/24h [20]. Consistent with the results of proteinuria, we also showed that RR for all-grade hypertension was 3.595 (95% CI: 2.952–4.378) and for high-grade hypertension was 5.173 (95% CI: 4.188–6.390). Now, hypertension resulted from bevacizumab was managed by oral antihypertensive medications [83]. In clinical practice, β-adrenoceptor antagonists, angiotensin converting enzyme inhibitors, angiotensin-receptor antagonists, and calcium antagonists may be used either in alone or in combination [84]. Besides, it was also recommended to temporary/permanent suspension or even hospitalization when severe hypertension could not be controlled by medications [19]. Above all, in order to properly manage hypertension and proteinuria, we should fully understand the pathogenesis of bevacizumab-associated renal toxicities and then select suitable therapeutic schemes in clinical practice.

Hypertension induced by bevacizumab may involve multiple reasons. Firstly, appropriate VEGF produced by podocytes could activate VEGF receptor on glomerular capillary endothelial cells to maintain the normal structure and function [85]. Whereas, bevacizumab, the VEGF inhibitor, would increase cell apoptosis and decrease endothelial renewal capacity [86]. Secondly, bevacizumab may suppress the production of vasodilators such as nitrous oxide and prostacyclin, which may in turn lead to vasoconstriction and decreased sodium ion renal excretion [87, 88]. Beside, bevacizumab may decrease the number of arterioles and capillaries, resulting in an increase in peripheral vascular resistance [89]. All of the above may be the explanations for bevacizumab related hypertension.

The pathogenesis for proteinuria induced by bevacizumab may also attribute to several pathways. First of all, bevacizumab could reduce proliferation of podocytes and endothelial cells due to the decreased renewal capacity [90]. Those proliferative changes would reduce the selection of protein filtration, which may lead to various levels of proteinuria and other clinical symptoms [91, 92]. In addition, hypertension induced by bevacizumab could increase intraglomerular pressure, thereby resulting in much more protein filtration [55]. But it was still unclear whether hypertension lead to proteinuria or both were resulted from bevacizumab independently.

Further stratified analyses based on various clinical characters were conducted to explore the confounding bias for the increased risks. Firstly, our meta-analysis suggested that patients accepted high-dose of bevacizumab at 5 mg/kg/ week had nearly double RR than those received low-dose at 2.5 mg/kg/week, which indicated that the increased risks of hypertension and proteinuria were dose-dependent. So, we could reduce the dosage to decrease the risks when bevacizumab must be used. Secondly, our study also showed that the risks for high-grade proteinuria and hypertension varied with tumor types, with the particularly highest risk for rental cancer. The explanation for this phenomenon may be that nephrectomy conducted among rental cancer patients could decrease glomerular filtration, which may lead to an underlying renal insufficiency. Consequently, a higher concentration of bevacizumab would aggravate the relative risks [93]. It was also possible that the hypertrophy of the postnephrectomy glomerular for rental cancer patients may become more dependence on VEGF to maintain structural integrity than a normal kidney, resulting in more susceptibility to bevacizumab [21]. Thirdly, stratified by concomitant drags, we found that the risks of proteinuric and hypertension may obvious augment when used in conjunction with interferonalfa, anthracycline or capecitabine. So, oncologists should be cautious to choose the relative lower toxicity concomitant drags for patients accepted bevacizumab. Whereas, no significant difference were discovered when stratified based on phase of trials, age of patients, treatment line and duration. So these factors should not be as the major considerations in the use of bevacizumab. Above all, clinicians should pay more attention when adding bevacizumab for the treatment of various cancers.

In conclusion, our study showed that bevacizumab significantly increased the risks of proteinuria and hypertension for cancer patients. Those risks may be dependent on dosage and vary with tumor types and concomitant drugs. So, early monitor and effective management of the risks may play vital role in more extensive and safer use of bevacizumab in clinical. Besides, future more studies were strongly encouraged to uncover the mechanisms of bevacizumab induced hypertension and proteinuria, and then guided therapy for these adverse effects.

## MATERIALS AND METHODS

### Identification and eligibility of relevant studies

Pubmed, Embase, Medline, and the Chinese Biomedical (CBM) databases were extensive searched using several search terms: “anti-VEGF antibody”, “bevacizumab”, “avastin”, “cancer”, “tumor”, “chemotherapy”, “adverse effects”, “proteinuria”, “hypertension”, “randomized controlled trials” and “RCTs” (last search updated on October, 2016). Additional literatures were identified by a handed search of the references of the original studies and reviews. In order to ensure that no clinical trials were overlooked, we also performed an independent search using the citation database Web of Science developed by the institute for scientific information. In the event that studies featured duplicate data, we incorporated all of the studies with various chemotherapy drugs and different follow-up time. If the relevant data were not freely available, we tried our best to contact investigators. Finally, we obtained further pertinent information by reviewing the updated manufacturer's package insert of bevacizumab.

### Study selection

Two investigators independently executed the literature search and examined RCTs to accurately assess the contribution of bevacizumab to the development of hypertension and proteinuria. Phase I trials and single-arm phase II trials were excluded from our present analysis. At last, the studies meeting the following criteria were selected for our meta-analysis; 1) prospective phase II or III RCTs, 2) random assignment of patients to either a case group combined treatment of bevacizumab and concurrent chemotherapy or a control group with chemotherapy alone, and 3) available data including the number of patients with hypertension or proteinuria for case and control groups (both high-grade or all-grade). Finally, 72 studies were included in our analysis and quality was assessed using previously described criteria including adequate blinding of randomization, completeness of follow-up, and objectivity of outcome measurement [94].

### Data extraction

The following data from these selected trials were independently extracted by two reviewers: the first author's last name, year of publication, cancer types, trial phase, trial line, age, the follow-up time, treatment duration, the number of enrolled patients, the number of intervention and control patients, concurrent chemotherapy, and bevacizumab dose. Any discrepancies between reviewers were resolved by consensus. Both hypertension and proteinuria were recorded according to version1, 2, 3 or 4 of the National Cancer Institute's Common Terminology Criteria for Adverse Events (CTCAE, http://ctep.cancer.gov/reporting/ctc_archive.html), which have been widely used in cancer clinical trials [95]. And both versions were similar regarding hypertension and proteinuria.

### Statistical analysis

We carried out all statistical analyses by the version 2 comprehensive meta-analysis program (Biostat, Englewood, NJ) [96]. Tthe overall incidence, risk ratios (RRs), and 95% confidence intervals (95% CIs) of patients with hypertension and proteinuria for each trial were calculated. And due to a possible correlation between rental cancer and proteinuria, we also carried out a separate analysis for proteinuria without rental cancer. Choosing a fixed or random-effect model was based on the heterogeneity estimated by calculating both Cochran Q statistic and I^2^ [97]. If the *P* value of Cochran Q statistic < 0.05, indicating a lack of homogeneity across study, so the result was reported by a random-effect model. Otherwise, fixed-effect model was adopted. Furthermore, Begg's funnel plots and Egger's linear regression test were applied to assess the publication bias. A two-tailed *P* value < 0.05 was judged as statistically significant.

## SUPPLEMENTARY MATERIALS TABLE








