# Supplementary material for: Bevacizumab significantly increases the risks of hypertension and proteinuria in cancer patients: A systematic review and comprehensive meta-analysis
Source: Oncotarget. 2017 May 23;8(31):51492–506. doi: 10.18632/oncotarget.18190 (PMC5584263; doi:10.18632/oncotarget.18190)
Supplement: Supplementary file 1 [file oncotarget-08-51492-s001.pdf]

Bevacizumab significantly increases the risks of hypertension and proteinuria in cancer patients: A systematic review and comprehensive meta-analysis

Supplementary Materials

Supplementry Table 1: Baseline characteristics of randomized–controlled trials (RCTs) included in the meta-analysis (*n* = 42510). See Supplementry_Table_1

Supplementry Table 2: Stratified analysis of incidence and RRs of high-grade hypertension and proeinuria for cancer patients treated with bevacizumab. See Supplementry_Table_2

Supplementry Table 3: Stratified analysis of incidence and RRs of all-grade hypertension and proeinuria for cancer patients treated with bevacizumab. See Supplementry_Table_3
