# Supplementary material for: Bevacizumab significantly increases the risks of hypertension and proteinuria in cancer patients: A systematic review and comprehensive meta-analysis
Source: Oncotarget. 2017 May 23;8(31):51492–506. doi: 10.18632/oncotarget.18190 (PMC5584263; doi:10.18632/oncotarget.18190)
Supplement: Supplementary file 2 [file oncotarget-08-51492-s002.docx]

**Table I. Baseline characteristics of randomized–controlled trials (RCTs) included in the meta-analysis (n = 42510).**

| **Author** | **Year** | **Tumor**  **type** | **Trial**  **phase** | **Trial line** | **Mean**  **age**  **(Y)** | **Total follow-**  **up**  **(Mo)** | **Treat-**  **ment durations** | **No.**  **enrolled** | **No.**  **analysis** | **No in intervention/control** | **Concurrent chemotherapy** | **Bev dose**  **Mg/kg per wk** |
| --- | --- | --- | --- | --- | --- | --- | --- | --- | --- | --- | --- | --- |
| Kabbinavar, | 2003 | CRC | II | 1 | NA | NR | NR | 104 | 70 | 35/35 | Fluorouracil and leucovorin  (FU/LV) | 2.5 |
|  |  |  |  |  |  |  |  |  | 67 | 32/35 |  | 5 |
| Hurwitz | 2004 | CRC | III | 1 | 59.5/  59.2 | NR | 9.43 | 813 | 790 | 393/397 | Irinotecan, fluorouracil, and leucovorin (IFL) | 2.5 |
| Kabbinavar, | 2005 | CRC | II | 1 | 71.3/  70.7 | 14.8 | 7.2 | 209 | 204 | 100/104 | Bolus fluorouracil and leucovorin | 2.5 |
| Giantonio | 2007 | CRC | III | 2 | 62/  60.8 | 28 | 1.87 | 829 | 572 | 287/285 | Oxaliplatin, fluorouracil, and leucovorin（FOLFOX4） | 5 |
| Saltz | 2008 | CRC | III | 1 | 60/60 | 27.6 | 6.3 | 1401 | 1369 | 694/675 | capecitabine and oxaliplatin (XELOX) and FOLFOX4 | 2.5 |
| Hochster | 2008 | CRC | III | 1 | 61/62 | NR | NR | 373 | 360 | 213/147 | bolus FU and LV with oxaliplatin (bFOL), or capecitabine with oxaliplatin (CapeOx) | 2.5 |
| Allegra | 2009 | CRC | III | 1 | NA | 28.5 | 11.5 | 2710 | 2647 | 1326/1321 | Bolus, infusion, fluorouracil, leucovorin with oxaliplatin  (FOLFOX6) | 2.5 |
| HURWITZ | 2009 | CRC | III | 1 | 62/58 | NR | NR | 230 | 229 | 128/101 | IFL | 2.5 |
| Tebbutt | 2010 | CRC | III | 1 | 67/69 | 31 | NR | 471 | 313 | 157/156 | Capecitabine | 2.5 |
| Statopoulos | 2010 | CRC | III | 1 | 67/62 | 36 | NR | 222 | 222 | 114/108 | Leucovorin, 5-fluorouracil plus irinotecan | 2.5 |
| Guan | 2011 | CRC | III | 1 | 53/50 | NR | 6.2 | 214 | 211 | 141/70 | Irinotecan, leucovorin bolus, fluorouracil, intravenous and infusion (mIFL) | 2.5 |
| Price | 2012 | CRC | III | 2 | NA | NR | NR | 471 | 471 | 315/156 | Capecitabine | 2.5 |
| Dotan | 2012 | CRC | II | 1 | 59/58 | 25.9 | NR | 32 | 23 | 12/11 | Oxaliplatin cetuximab IV | 2.5 |
| De Gramont | 2012 | CRC | III | 1 | 58/58 | 48.3 | 10.6 | 3451 | 2271 | 1145/1126 | FOLFOX4 | 2.5 |
| Infante | 2013 | CRC | II | 1 | 59/61 | NR | NR | 126 | 80 | 41/39 | Infused, fluorouracil, folinic acid, oxaliplatin and Axitinib  (FOLFOX ) | 2.5 |
| Allegra | 2013 | CRC | III | 1 | NA | 58.8 | NR | 2710 | 2673 | 1335/1338 | Oxaliplatin | 2.5 |
| Bennouna | 2013 | CRC | III | 2 | 63/63 | 11.1 | 3.9 | 820 | 810 | 401/409 | Bolus fluorouracil capecitabine oxaliplatin irinotecan(XELIRI) | 2.5 |
| Cunningham | 2013 | CRC | III | 1 | 76/77 | 24.8 | 5.8 | 280 | 270 | 134/136 | Capecitabine | 2.5 |
| Cao | 2015 | CRC | II | 2 | 62/61 | NR | NR | 142 | 142 | 65/77 | Irinotecan, fluorouracil, and leucovorin (FOLFIRI) | 5 |
| Susanna | 2015 | CRC | III | 1 | 65/66 | 17 | NR | 472 | 314 | 156/158 | NA | 2.5 |
| Passardi | 2015 | CRC | III | 1 | 66/66 | 36 | NR | 376 | 370 | 176/194 | FOLFIRI and FOLFOX4 | 2.5 |
| **Author** | **Year** | **Tumor type** | **Trial phase** | **Trial line** | **Mean**  **age**  **(Y)** | **Total follow-**  **up**  **(mo)** | **Treat-**  **ment durations** | **No.**  **enrolled** | **No.**  **analysis** | **No in intervention/control** | **Concurrent chemotherapy** | **Bev dose**  **Mg/kg per wk** |
| Miller | 2005 | BC | III | 2 | 51/52 | 14.8 | NR | 462 | 444 | 229/215 | Capecitabine | 5 |
| Miller | 2007 | BC | III | 1 | 56/55 | 25.9 | 7.1 | 722 | 711 | 365/346 | Paclitaxel | 5 |
| Miles | 2008 | BC | III | 1 | 54/55 | 10.2 | NR | 736 | 483 | 252/231 | Docetaxel | 2.5 |
|  |  |  |  |  | 55/55 |  |  |  | 478 | 247/231 | Docetaxel | 5 |
| Martin | 2011 | BC | II | 1 | 55/53 | 60 | NR | 282 | 185 | 96/89 | Paclitaxel | 5 |
| Brufsky | 2011 | BC | III | 2 | 55/55 | 15 | 6 | 684 | 679 | 458/221 | Capecitabine, taxane, gemcitabine, or vinorelbine | 5 |
| Robert | 2011 | BC | III | 1 | 55/56 | 15.6 | NR | 1237 | 605 | 404/201 | Capecitabine | 5 |
|  |  |  |  |  |  | 19.2 |  |  | 305 | 203/102 | Taxane |  |
|  |  |  |  |  |  | 19.2 |  |  | 310 | 210/100 | Anthracycline |  |
| von Minckwitz | 2012 | BC | III | 1 | 49/48 | NR | NR | 1948 | 1925 | 956/969 | Epirubicin, cyclophosphamide and docetaxel | 5 |
| Bear | 2012 | BC | III | 1 | NA | 33 | NR | 1206 | 1191 | 595/596 | Docetaxel, capecitabine and gemcitabine | 5 |
| Gilles | 2013 | BC | III | 1 | 53/55 | 26 | 11.7 | 424 | 421 | 215/206 | Docetaxel and trastuzumab | 5 |
| Cameron | 2013 | BC | III | 1 | 50/50 | 32 | NR | 2591 | 2559 | 1288/1271 | Anthracycline, taxane, or both | 5 |
| Coudert | 2014 | BC | II | 1 | 50/47 | NR | NR | 152 | 72 | 47/25 | Trastuzumab and docetaxel | 5 |
| Bear | 2015 | BC | III | 1 | NA | 72 | NR | 1206 | 1190 | 594/596 | Docetaxel capecitabine gemcitabine doxorubicin and cyclophosphamide | 5 |
| Earl | 2015 | BC | III | 1 | NA | 36 | NR | 800 | 775 | 384/391 | Docetaxel fluorouracil epirubicin cyclophosphamide (D-FEC) | 5 |
| Sikov | 2015 | BC | III | 1 | NA | NR | NR | 454 | 225 | 112/113 | Carboplatin doxorubicin and cyclophosphamide | 5 |
| Nahleh | 2016 | BC | II | 1 | 51.7/  51.3 | 36 | NR | 215 | 205 | 95/110 | Doxorubicin, cyclophosphamide and nab-paclitaxel intravenous | 5 |
| Johnson | 2004 | LC | II | 1 | NA | 14.7 | 6.3 | 99 | 64 | 32/32 | Carboplatin and paclitaxel | 2.5 |
|  |  |  |  |  |  |  |  |  | 66 | 34/32 | Carboplatin and paclitaxel | 5 |
| Sandler, | 2006 | LC | III | 1 | NA | 19 | 4.9 | 878 | 867 | 427/440 | Paclitaxel and carboplatin | 5 |
| Herbst | 2007 | LC | II | 1 | 63/65 | 15.8 | NR | 122 | 81 | 39/42 | Docetaxel or pemetrexed | 5 |
| Reck | 2009 | LC | III | 1 | 57/59 | NR | 4.4 | 1043 | 657 | 330/327 | Cisplatin and gemcitabine | 2.5 |
|  |  |  |  |  | 59/59 |  |  |  | 656 | 329/327 |  | 5 |
| Spigel | 2011 | LC | II | 1 | 60/64 | 8.1 | NR | 102 | 98 | 51/47 | Cisplatin, carboplatin and etoposide | 5 |
| Soria | 2011 | LC | II | 1 | 58/62 | NR | NR | 213 | 83 | 42/41 | Paclitaxel and carboplatin | 5 |
| Herbst | 2011 | LC | III | 2 | 65/65 | NR | 2.8 | 636 | 626 | 313/313 | Erlotinib | 5 |
| MOK | 2011 | LC | III | 1 | 57/57 | NR | NR | 105 | 66 | 35/31 | Cisplatin and gemcitabine | 2.5 |
|  |  |  |  |  |  |  |  |  | 64 | 33/31 |  | 5 |
| Niho | 2012 | LC | II | 1 | 61/60 | NR | NR | 180 | 177 | 119/58 | Carboplatin-paclitaxel | 5 |
| **Author** | **Year** | **Tumor type** | **Trial phase** | **Trial line** | **Mean**  **age**  **(Y)** | **Total follow**  **up**  **(mo)** | **Treat-**  **ment durations** | **No.**  **enrolled** | **No.**  **analysis** | **No in intervention/control** | **Concurrent chemotherapy** | **Bev dose**  **Mg/kg per wk** |
| Boutsikou | 2013 | LC | III | 1 | 62/65 | 48 | NR | 229 | 112 | 60/52 | Docetaxel, carboplatin erlotinib | 2.5 |
| Seto | 2014 | LC | II | 1 | 67/67 | 20.4 | NR | 154 | 152 | 75/77 | Erlotinib | 5 |
| Zhou | 2015 | LC | III | 1 | 57/56 | 28.1 | NR | 276 | 274 | 140/134 | Carboplatin and paclitaxel | 5 |
| Pujol | 2015 | LC | III | 1 | 61.2/  60.1 | 24 | NR | 147 | 74 | 37/37 | Cisplatin, epidoxorubicin, etoposide, cyclophosphamide | 2.5 |
| Takeda | 2016 | LC | II | 2 | 64/67 | 11.2 | NR | 100 | 100 | 50/50 | Docetaxel | 5 |
| Karayama | 2016 | LC | II | 1 | 65/66 | 24.1 | NR | 110 | 80 | 35/45 | Pemetrexed | 5 |
| Yang | 2003 | RCC | II | 1 | 53/54 | 27 | NR | 116 | 77 | 37/40 | None | 2.5 |
|  |  |  |  |  |  |  |  |  | 79 | 39/40 |  | 5 |
| Escudier | 2007 | RCC | III | 1 | 61/60 | 13.3 | 9.7 | 649 | 641 | 337/304 | Interferonalfa | 5 |
| Rini | 2008 | RCC | III | 1 | NA | 46.2 | 5.7 | 732 | 709 | 362/347 | Interferonalfa | 5 |
| Cutsem | 2009 | PC | III | 1 | 62/61 | NR | NR | 607 | 583 | 296/287 | Gemcitabine erlotinib | 2.5 |
| Kindler | 2010 | PC | III |  | 64/65 | NR | 4.1 | 602 | 540 | 277/263 | Gemcitabine | 5 |
| Burger | 2011 | OC | III | 1 | 60/60 | 17.4 | NR | 1873 | 1217 | 608/601 | Carboplatin paclitaxel | 5 |
| Perren | 2011 | OC | III | 1 | NA | 19.4 | NR | 1528 | 1498 | 745/753 | Carboplatin paclitaxel | 2.5 |
| Pujade | 2012 | OC | III | 2 | 62/61 | 13.9 | NR | 361 | 360 | 179/181 | Paclitaxel PLD topotecan | 5 |
| Aghajanian | 2015 | OC | III | 1 | 60.5/  61.6 | 58.2 | 8.4 | 484 | 480 | 247/233 | Gemcitabine and carboplatin | 5 |
| Ohtsu | 2011 | GC | III | 1 | 58/59 | 7 | 6.8 | 774 | 767 | 386/381 | cisplatin capecitabine | 2.5 |
| Okines | 2013 | GC | II | 2 | 64/62 | 22 | NR | 213 | 200 | 99/101 | Epirubicin, cisplatin and capecitabine | 2.5 |
| Chinot | 2014 | Glioblastoma | III | 2 | 57/56 | NR | NR | 921 | 911 | 461/450 | Radiotherapy–temozolomide | 5 |
| Walter | 2014 | Glioblastoma | II | 2 | 55/56 | 35.3 | NR | 153 | 98 | 52/46 | Lomustine | 5 |
| Seymour | 2014 | Lymphoma | III | 1 | 61/61 | 23.7 | NR | 787 | 781 | 395/386 | Rituximab, cyclophosphamide, doxorubicin, vincristine, and prednisone(R-CHOP) | 5 |
| Hainsworth | 2014 | Lymphoma | II | 2 | 68/65 | 34 | NR | 60 | 60 | 29/31 | Rituximab | 5 |
| Kim | 2012 | Melanoma | II | 1 | 60/60 | 13 | 4.2 | 214 | 212 | 143/69 | Carboplatin plus paclitaxel | 5 |
| White | 2013 | Myeloma | II | 2 | 65/65 | 13.3 | 4.2 | 102 | 100 | 50/50 | bortezomib | 5 |
| Kindler | 2012 | Malignant  mesothelioma | II | 1 | 62/65 | 15.6 | 4.9 | 115 | 108 | 53/55 | Cisplatin gemcitabine | 5 |
| Kelly | 2012 | Prostate  cancer | III | 1 | 68.8/  69.3 | 25 | 5.6 | 1050 | 1009 | 504/  505 | docetaxel | 5 |
| Tewari | 2014 | Cervical  cancer | III | 2 | NA | 20.8 | NR | 452 | 439 | 220/219 | Cisplatin paclitaxel topotecan and paclitaxel | 5 |
| Hensley | 2015 | Leiomyo  sarcoma | III | 1 | 54.8/  56.2 | 26.9 | NR | 107 | 103 | 52/  51 | Gemcitabine docetaxel | 5 |

**Abbreviations:** Bev, Bevacizumab; CRC, Colorectal cancer; BC, Breast cancer; LC, Lung cancer; RCC, Rental cancer; PC, Pancreatic cancer; OC, Ovarian cancer; GC, Gastric cancer.
