# Supplementary material for: Bevacizumab significantly increases the risks of hypertension and proteinuria in cancer patients: A systematic review and comprehensive meta-analysis
Source: Oncotarget. 2017 May 23;8(31):51492–506. doi: 10.18632/oncotarget.18190 (PMC5584263; doi:10.18632/oncotarget.18190)
Supplement: Supplementary file 3 [file oncotarget-08-51492-s003.docx]

**Table II. Stratified analysis of incidence and RRs of high-grade hypertension and proeinuria for cancer patients treated with bevacizumab.**

|  | **High-grade** **hypertension** | | | | | **High-grade proeinuria** | | | | |
| --- | --- | --- | --- | --- | --- | --- | --- | --- | --- | --- |
|  |  | **Bev** | **Control** |  |  |  | **Bev** | **Control** |  |  |
| **Categories** | **No. of studies** | **(Events/**  **total)** | **(Events/**  **total)** | **Incidence**  **(%,95%CI)** | **RR(95%CI)** | **No. of studies** | **(Events/**  **total)** | **(Events/**  **total)** | **Incidence**  **(%,95%CI)** | **RR(95%CI)** |
| **Overall** | 66 | 1677/  20171 | 246/  18848 | 8.2%(7-9.8) | 5.173(4.188-6.390) | 45 | 375/  15522 | 30/  14384 | 2.4(1.8-3.2) | 5.494(3.991-7.564) |
| **Bevacizumab dose** | |  |  |  |  |  |  |  |  |  |
| **2.5mg/**  **kg per wk** | 23 | 504/  8987 | 88/  8600 | 5.2(3.9-6.9) | 3.875(2.645-5.675) | 17 | 82/6591 | 12/6301 | 1.4(0.9-2.1) | 4.096(2.350-7.139) |
| **5mg/**  **kg per wk** | 45 | 1173/  11184 | 158/  10248 | 10.5(8.7-12.7) | 6.020(4.661-7.775) | 31 | 293/  8931 | 18/  8083 | 3.2(2.3-4.4) | 6.354(4.298-9.394) |
| **tumor types** | |  |  |  |  |  |  |  |  |  |
| **CRC** | 18 | 427/  7099 | 81/  6765 | 6.4(4.6-8.9) | 3.703(2.480-5.531) | 12 | 69/  4798 | 11/  4543 | 1.7(1-2.8) | 4.063(2.189-7.544) |
| **BC** | 14 | 499/  5794 | 37/  5041 | 8.4(6.7-10.5) | 7.621(4.028-14.419) | 8 | 71/  4255 | 2/  3515 | 1.8(1.2-2.8) | 7.483(3.326-16.834) |
| **LC** | 14 | 186/  2139 | 31/  2075 | 8.2(4.9-13.4) | 4.968(3.471-7.110) | 7 | 36/  1538 | 0/  1489 | 2.9(1.7-5) | 6.813(2.512-18.478) |
| **RCC** | 3 | 58/  738 | 2/  691 | 9.1(3.6-21.3) | 13.074(2.631-64.96) | 3 | 83/  775 | 1/  731 | 8.9(4.6-16.5) | 22.786(6.347-81.804) |
| **PC** | 2 | 40/  573 | 11/  550 | 6.3(1.9-18.5) | 3.472(1.804-6.679) | 2 | 21/  573 | 3/  550 | 3.6(1.7-7.5) | 5.343(1.719-16.606) |
| **OC** | 4 | 243/  1779 | 49/  178 | 12.1(5.9-23.3) | 8.981(2.813-28.678) | 4 | 44/  1779 | 7/  1768 | 2.1(0.5-8.9) | 4.831(2.100-11.110) |
| **GC** | 2 | 25/  485 | 2/  482 | 3.3(0.6-16.4) | 9.429(2.547-34.910) | 2 | 3/  485 | 0/  482 | 0.6(0.2-2) | 3.933(0.437-35.412) |
| **Glioblastoma** | 2 | 66/  513 | 13/  496 | 17.3(6.9-37.1) | 4.830(2.707-8.619) | 2 | 28/  513 | 0/  496 | 5.5(3.5-7.8) | 18.492(2.410-141.95) |
| **Others** | 7 | 133/  1051 | 20/  980 | 13.1(6.4-24.8) | 5.542(3.071-10.000) | 4 | 20/  806 | 6/  810 | 3.2(1.1-9) | 2.990(1.217-7.346) |
| **Phase of trials** | |  |  |  |  |  |  |  |  |  |
| **Phase II** | 21 | 192/  1411 | 43/  1289 | 11.8(7.6-17.8) | 3.387(2.403-4.774) | 12 | 31/  794 | 5/  763 | 5.1(3.4-7.7) | 3.181(1.504-6.727) |
| **Phase III** | 45 | 1485/  18760 | 203/  17559 | 7.2(6-8.6) | 5.874(4.854-7.528) | 33 | 344/  14728 | 25/  13621 | 2.1(1.4-2.9) | 6.206(4.357-8.837) |
| **Treatment line** | |  |  |  |  |  |  |  |  |  |
| **First line** | 51 | 1383/  16963 | 205/  16044 | 8.1(6.7-9.8) | 5.182(4.055-6.623) | 33 | 300/  13078 | 27/  12352 | 2.3(1.6-3.3) | 5.351(3.771-7.592) |
| **Second line** | 15 | 294/  3208 | 41/  2804 | 9(6.2-12.9) | 5.086(3.393-7.624) | 12 | 75/  2444 | 3/  2032 | 3(2.1-4.4) | 6.282(2.857-13.809) |
|  | **High-grade hypertension** | | | | | **High-grade proeinuria** | | | | |
|  |  | **Bev** | **Control** |  |  |  | **Bev** | **Control** |  |  |
| **Categories** | **No. of studies** | **(Events/**  **total)** | **(Events/**  **total)** | **Incidence**  **(%,95%CI)** | **RR(95%CI)** | **No. of studies** | **(Events/**  **total)** | **(Events/**  **total)** | **Incidence**  **(%,95%CI)** | **RR(95%CI)** |
| **Concomitant drugs** | |  |  |  |  |  |  |  |  |  |
| **Taxane** | 21 | 469/  4725 | 76/  4398 | 7.4(5.2-10.2) | 5.407(3.418-8.553) | 18 | 84/  4274 | 8/  4057 | 2.1(1.6-2.9) | 4.793(2.650-8.670) |
| **Oxaliplatin** | 7 | 294/  5042 | 50/  4931 | 6.1(3.5-10.4) | 3.888(1.875-8.060) | 4 | 39/  3493 | 5/  3446 | 1.2(0.7-2) | 6.173(2.593-14.698) |
| **Cyclophosphamide** | 5 | 89/  1222 | 11/  1244 | 6.3(3.4-11.4) | 5.701(1.417-22.947) | 5 | 1/  384 | 0/  391 | 0.3(0-1.8) | 3.055(0.125-74.750) |
| **Gemcitabine** | 8 | 163/  1600 | 32/  1554 | 10.9(6.3-18.4) | 4.170(2.809-6.190) | 7 | 61/  1565 | 7/  1523 | 3.9(1.9-8) | 5.882(2.906-11.909) |
| **Capecitabine** | 7 | 131/  1724 | 9/  1346 | 6.1(3.5-10.3) | 8.028(3.884-16.694) | 3 | 36/  1724 | 2/  1346 | 2(1.1-3.5) | 5.630(2.086-15.194) |
| **Irinotecan** | 4 | 71/  727 | 17/  654 | 9.6(6.1-14.7) | 3.783(2.259-6.333) | 3 | 8/  599 | 3/  545 | 1.7(0.3-7.7) | 1.704(0.474-6.128) |
| **Anthracycline** | 2 | 107/  1498 | 5/  1371 | 8.1(5.1-12.5) | 17.174(7.301-40.398) | 1 | 14/  1498 | 1/  1371 | 1.3(0.3-5.7) | 7.273(1.353-39.110) |
| **Interferon alfa** | 2 | 50/  699 | 2/  651 | 6.1(1.8-18.6) | 14.898(1.084-204.77) |  | 78/  699 | 1/  651 | 10.3(4.3-22.8) | 48.931(9.763-245.31) |
| **Treatment duration** | |  |  |  |  |  |  |  |  |  |
| **<6.2 momths** | 13 | 304/  4068 | 54/  3747 | 7.7(5.6-10.7) | 4.192(2.958-5.942) | 10 | 122/  3161 | 10/  2906 | 2.6(1.2-5.3) | 5.784(3.160-10.588) |
| **>6.2 momths** | 12 | 450/  5415 | 50/  5227 | 8.2(6.0-11.1) | 7.045(4.556-10.894) | 11 | 106/  5349 | 10/  5164 | 1.7(0.8-3.4) | 5.786(2.746-12.189) |
| **No reported** | 41 | 923/  10688 | 142/  9874 | 8.5(6.7-10.8) | 5.000(3.715-6.729) | 24 | 147/  7012 | 10/  6314 | 2.6(1.9-3.5) | 5.128(3.192-8.240) |
| **Age (Year)** | |  |  |  |  |  |  |  |  |  |
| **<60 years** | 23 | 709/  7814 | 80/  6899 | 9.3(7.9-10.9) | 5.774(4.031-8.269) | 18 | 134/  7398 | 7/  6533 | 2.1(1.5-3) | 5.618(3.254-9.694) |
| **>60 years** | 30 | 538/  5809 | 123/  5548 | 8.0(5.6-11.4) | 3.690(3.054-4.459) | 20 | 127/  4345 | 17/  4224 | 2.9(1.9-4.5) | 4.401(2.772-6.986) |
| **No reported** | 13 | 430/  6548 | 43/  6401 | 7.1(4.9-10.3) | 7.417(3.881-14.174) | 7 | 114/  3779 | 6/  3627 | 2.2(0.8-6) | 9.564(4.481-20.414) |

**Abbreviations:** Bev, Bevacizumab; CRC, Colorectal cancer; BC, Breast cancer; LC, Lung cancer; RCC, Rental cancer; PC, Pancreatic cancer; OC,Ovarian cancer; GC, Gastric cancer; Others including Lymphoma, Melanoma, Malignant mesothelioma, Prostate cancer, Cervical cancer, Leiomyosarcoma.
