# Supplementary material for: Bevacizumab significantly increases the risks of hypertension and proteinuria in cancer patients: A systematic review and comprehensive meta-analysis
Source: Oncotarget. 2017 May 23;8(31):51492–506. doi: 10.18632/oncotarget.18190 (PMC5584263; doi:10.18632/oncotarget.18190)
Supplement: Supplementary file 4 [file oncotarget-08-51492-s004.docx]

**Table III. Stratified analysis of incidence and RRs of all-grade hypertension and proeinuria for cancer patients treated with bevacizumab.**

|  | **All-grade hypertension** | | | | | **All-grade proeinuria** | | | | |
| --- | --- | --- | --- | --- | --- | --- | --- | --- | --- | --- |
|  |  | **Bev** | **Control** |  |  |  | **Bev** | **Control** |  |  |
| **Categories** | **No. of studies** | **(Events/**  **total)** | **(Events/**  **total)** | **Incidence**  **(%,95%CI)** | **RR(95%CI)** | **No. of studies** | **(Events/**  **total)** | **(Events/**  **total)** | **Incidence**  **(%,95%CI)** | **RR(95%CI)** |
| **Overall** | 39 | 2066/  9613 | 519/  9444 | 25.3(21.5-29.5) | 3.595(2.952-4.378) | 27 | 1146/  8358 | 341/  8234 | 18(11.7-26.6) | 3.369(2.492-4.556) |
| **Bevacizumab dose** | |  |  |  |  |  |  |  |  |  |
| **2.5mg/**  **kg per wk** | 18 | 696/  3886 | 214/  891 | 22.4(16.6-29.5) | 2.969(2.311-3.815) | 14 | 353/  3651 | 196/  3655 | 12.4(6.9-21.3) | 2.124(1.557-2.897) |
| **5mg/**  **kg per wk** | 25 | 1370/  5727 | 305/  5553 | 27.3(22.4-32.7) | 4.068(3.067-5.397) | 15 | 793/  4707 | 145/  4579 | 24.6(13.3-41) | 4.225(2.923-6.106) |
| **Tumor types** | |  |  |  |  |  |  |  |  |  |
| **CRC** | 12 | 434/  2963 | 134/  2979 | 22.2(14.7-32.1) | 3.181(2.316-4.368) | 9 | 282/  2728 | 158/  2740 | 11.9(5.7-23.3) | 2.324(1.551-3.484) |
| **BC** | 7 | 657/  3233 | 123/  3183 | 21.1(17.3-25.3) | 5.119(2.415-10.849) | 4 | 200/  2857 | 41/  2846 | 8.9(3.8-19.8) | 4.142(2.184-7.875) |
| **LC** | 10 | 220/  591 | 53/  528 | 31.5(20.6-44.9) | 3.337(2.464-4.519) | 7 | 158/  444 | 27/  381 | 26.8(15.1-43) | 4.074(2.689-6.172) |
| **RCC** | 3 | 206/  775 | 45/  731 | 27.2(20.8-34.7) | 4.062(1.804-9.149) | 3 | 356/  775 | 62/  731 | 47.1(17.3-79.1) | 3.348(1.086-10.32) |
| **PC** | 1 | 60/296 | 26/287 | 20.3(16.1-25.2) | 2.238(1.455-3.442) | 1 | 15/296 | 4/287 | 5.1(3.1-8.2) | 3.636(1.221-10.82) |
| **OC** | 1 | 193/  745 | 47/  753 | 25.9(22.9-29.2) | 4.150(3.068-5.615) | 1 | 33/  745 | 19/  753 | 4.4(3.2-6.2) | 1.755(1.008-3.059) |
| **Glioblastoma** | 2 | 211/  513 | 68/  496 | 47.3(30.2-6.9) | 2.961(2.324-3.771) | 2 | 102/  513 | 30/  496 | 33.1(6.5-77.8) | 3.062(2.020-4.642) |
| **Others** | 3 | 85/  497 | 23/  487 | 17.2(14.1-20.8) | 3.129(1.349-7.260) | NR | NR | NR | NR | NR |
| **Phase of trials** | |  |  |  |  |  |  |  |  |  |
| **Phase II** | 17 | 340/  967 | 94/  891 | 30.8(22.7-40.2) | 3.134(2.329-4.216) | 8 | 264/  548 | 91/  499 | 48.4(41.8-55.1) | 2.579(1.782-3.734) |
| **Phase III** | 24 | 1726/  8646 | 425/  8553 | 21.7(18-25.9) | 3.795(2.954-4.875) | 19 | 882/  7810 | 250/  7735 | 10.1(5.8-17.1) | 3.983(2.597-6.109) |
| **Treatment line** | |  |  |  |  |  |  |  |  |  |
| **First line** | 33 | 1752/  8484 | 427/  8348 | 24.9(21-29.2) | 3.662(2.923-4.587) | 22 | 968/  7279 | 286/  7188 | 17.6(10.6-27.9) | 3.574(2.474-5.164) |
| **Second line** | 6 | 314/  1129 | 92/  1096 | 28(16-44.2) | 3.219(2.113-4.903) | 5 | 178/  1078 | 55/  1046 | 20.7(9.5-39.3) | 2.973(2.259-3.912) |
| **Concomitant drugs** | |  |  |  |  |  |  |  |  |  |
| **Taxane** | 10 | 329/  1264 | 83/  1174 | 21.4(14.9-29.9) | 3.115(2.256-4.302) | 8 | 122/  974 | 38/  913 | 19.3(3.6-60.2) | 2.381(1.690-3.354) |
|  | **All-grade hypertension** | | | | | **All-grade proeinuria** | | | | |
|  |  | **Bev** | **Control** |  |  |  | **Bev** | **Control** |  |  |
| **Categories** | **No. of studies** | **(Events/**  **total)** | **(Events/**  **total)** | **Incidence**  **(%,95%CI)** | **RR(95%CI)** | **No. of studies** | **(Events/**  **total)** | **(Events/**  **total)** | **Incidence**  **(%,95%CI)** | **RR(95%CI)** |
| **Oxaliplatin** | 5 | 129/  1666 | 32/  1656 | 22.2(5.6-57.7) | 3.405(1.351-8.580) | 1 | 35/  1654 | 6/  1645 | 3.6(0.3-30.4) | 5.113(2.263-11.55) |
| **Cyclophosphamide** | 5 | 282/  1410 | 70/  1410 | 22.7(15.5-32) | 4.290(1.439-12.790) | 4 | 110/  1377 | 10/  1397 | 8.7(4-18.1) | 11.515(2.106-62.9) |
| **Gemcitabine** | 1 | 89/  364 | 36/  349 | 33.4(15.4-58) | 2.350(1.645-3.358) | 1 | 22/  364 | 4/  349 | 7(4-11.9) | 4.236(1.617-11.1) |
| **Capecitabine** | 3 | 126/  520 | 31/  507 | 24.2(19.3-29.8) | 4.225(1.874-9.527) | 3 | 110/  520 | 36/  507 | 18.8(9.7-33.2) | 2.857(2.011-4.058) |
| **Irinotecan** | 4 | 111/  507 | 33/  505 | 21.9(18.5-25.7) | 7.686(0.537-109.921) | 2 | 111/  507 | 86/  505 | 13.8(2.9-45.9) | 2.717(0.285-25.89) |
| **Anthracycline** | 2 | 224/  1288 | 41/  1271 | 17.4(15.4-19.6) | 5.391(3.9-7.453) | 1 | 42/  1288 | 15/  1271 | 3.3(2.4-4.4) | 2.763(1.540-4.957) |
| **Interferon alfa** | 1 | 191/  699 | 41/  651 | 27.3(24.2-30.8) | 4.547(1.732-11.932) | 1 | 316/  699 | 32/  651 | 41.9(6.2-88.8) | 9.229(6.402-13.30) |
| **Treatment duration** | |  |  |  |  |  |  |  |  |  |
| **<6.2 momths** | 4 | 158/  833 | 27/  818 | 17.2(8.6-31.5) | 5.496(3.690-8.187) | 3 | 269/  783 | 25/  768 | 10.6(0.5-73.7) | 10.14(6.926-14.86) |
| **>6.2 momths** | 5 | 297/  2222 | 77/  2190 | 18.2(9.5-32) | 4.173(2.641-6.592) | 4 | 222/  2156 | 117/  2126 | 14.3(4.6-36.6) | 2.893(1.304-6.416) |
| **No reported** | 30 | 1611/  6558 | 415/  6436 | 28.2(24-32.9) | 3.358(2.657-4.242) | 20 | 655/  5419 | 199/  5340 | 19.7(12.8-29.2) | 2.908(2.265-3.733) |
| **Age (Year)** | |  |  |  |  |  |  |  |  |  |
| **<60 years** | 13 | 704/  2857 | 202/  2777 | 29.7(22.4-38.2) | 2.848(2.142-3.787) | 9 | 411/  3564 | 180/  3529 | 20.5(11.5-34.1) | 2.382(1.609-3.525) |
| **>60 years** | 18 | 620/  2617 | 181/  2529 | 26.1(19.9-33.4) | 3.163(2.564-3.901) | 14 | 370/  1977 | 105/  1893 | 18.8(11.6-29) | 3.406(2.431-4.773) |
| **No reported** | 8 | 742/  4139 | 136/  4138 | 18.6(13-25.8) | 6.166(3.316-11.469) | 4 | 365/  2817 | 56/  2812 | 11.7(1.4-55.5) | 5.06(2.024-12.649) |

**Abbreviations:** Bev, Bevacizumab; CRC, Colorectal cancer; BC, Breast cancer; LC, Lung cancer; RCC, Rental cancer; PC, Pancreatic cancer; OC,Ovarian cancer; GC, Gastric cancer; Others including Lymphoma, Melanoma, Malignant mesothelioma, Prostate cancer, Cervical cancer, Leiomyosarcoma.
